# Supplementary material for: Naringin Exhibited Therapeutic Effects against DSS-Induced Mice Ulcerative Colitis in Intestinal Barrier–Dependent Manner
Source: Molecules. 2021 Oct 31;26(21):6604. doi: 10.3390/molecules26216604 (PMC8588024; doi:10.3390/molecules26216604)
Supplement: Supplementary file 1 [file molecules-26-06604-s001.zip › molecules-1420210-supplementary.pdf]

Supporting Information

Naringin exhibited therapeutic effects against DSS-induced mice ulcerative colitis in intestinal barrier–dependent manner

Ruige Cao [1,†], Xing Wu [1,†], Hui Guo [1], Xin Pan [1], Rong Huang [1,*], Gangqiang Wang [1,2,*] and Jikai Liu [1,*]

1 School of Pharmaceutical Sciences, South-Central University for Nationalities, Wuhan 430074, China;

2 Hubei Key Laboratory of Radiation Chemistry and Functional Materials, Non-power Nuclear Technology Collaborative Innovation Center, Hubei University of Science and Technology, Xianning 437100, China;

*Corresponding author:

E-mail: ronghuang@mail.scuec.edu.cn (R.H.)

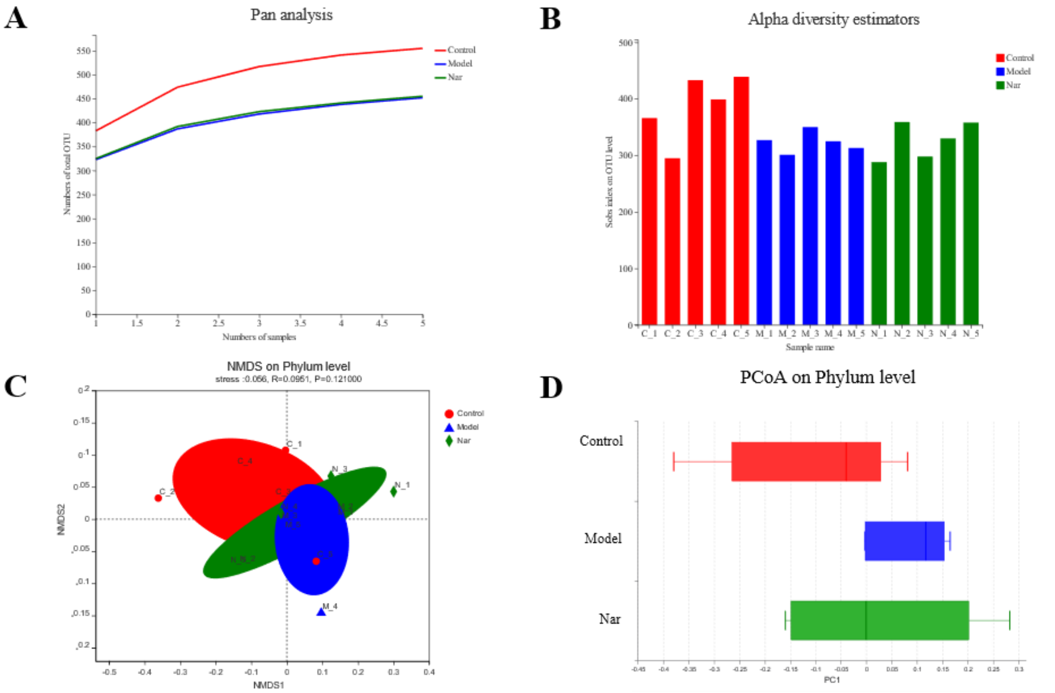

Figure S1. Naringin regulates the composition of intestinal flora in mice. (A) Pan/Core species analysis, (B) Alpha diversity analysis, (C) NMDS analysis, (D) PCoA analysis (n = 5).

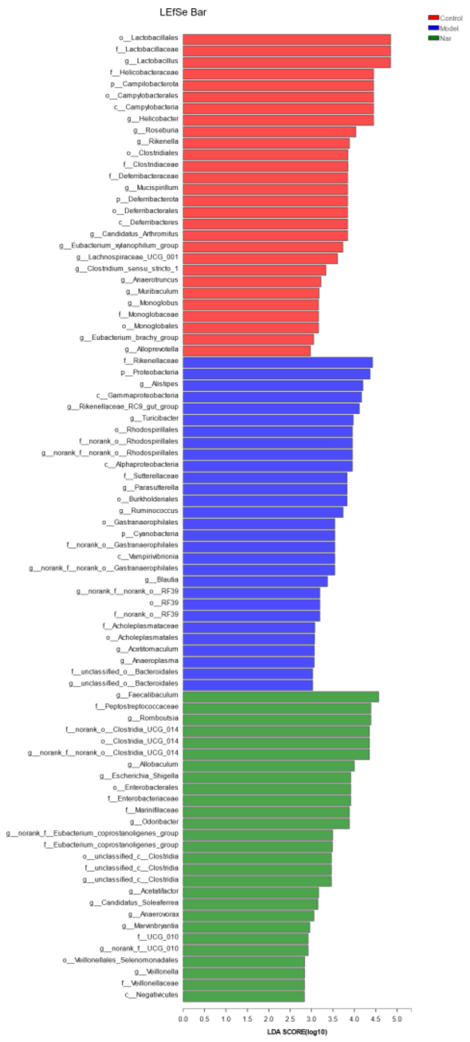

Figure S2. LDA analyses based on OTUs characterized the microbiomes of the control group, model group and Nar group. LDA scores show the significant bacterial differences between three groups (n = 5).
